# A Novel Supplement Consisting of Rice, Silkworm Pupae and a Mixture of Ginger and Holy Basil Improves Post-Stroke Cognitive Impairment

**DOI:** 10.3390/nu16234144

**Published:** 2024-11-29

**Authors:** Putthiwat Thongwong, Jintanaporn Wattanathorn, Wipawee Thukham-mee

**Affiliations:** 1 Department of Physiology and Graduate School (Neuroscience Program), Faculty of Medicine, Khon Kaen University, Khon Kaen 40000, Thailand; putthiwat.t@gmail.com; 2 Research Institute for High Human Performance and Health Promotion, Khon Kaen University, Khon Kaen 40000, Thailand; meewep@gmail.com; 3 Center of Excellence for Functional Food and Health Innovation Faculty of Medicine, Khon Kaen University, Khon Kaen 40000, Thailand; 4 Department of Physiology, Faculty of Medicine, Khon Kaen University, Khon Kaen 40000, Thailand

**Keywords:** post-stroke cognitive impairment, stress, orodispersible film, silkworm pupae hydrolysate, holy basil, ginger

## Abstract

**Backgrounds/Objectives:** Despite the increasing importance of the condition of post-stroke cognitive impairment (PSCI), the current therapy efficacy is limited. Since oxidative stress and inflammation are targeted in anti-stroke therapy, we aimed to assess the protective effect against PSI of an orodispersible film loaded with silkworm pupae hydrolysate and a combined extract of holy basil and ginger (JP1), which show antioxidant, and anti-inflammation effects. **Methods:** Male Wistar rats (200–250 g) were administered JP1 at doses of 1, 10, and 100 mg/kg BW 45 min before a 6 h immobilization stress exposure for 14 days. Then, the right middle cerebral artery was permanently occluded (MCAO) and JP1 was continually administered for 21 days after MCAO. Spatial and non-spatial memory and the possible underlying mechanisms were also explored. **Results:** JP1 improved oxidative stress, inflammation, apoptosis, Erk signaling pathway, cholinergic function, and the growth of *Lactobacillus* and *Bifidobacterium* spp. in feces. These results suggest that JP1 improves PSCI, possibly involving the above mechanisms. Furthermore, serum corticosterone also decreased. **Conclusions:** Our results suggest that JP1 is a potential candidate for combating PSCI following exposure to stroke plus stress. However, a clear understanding of the precise active ingredient and the detailed mechanisms require further investigation.

## 1. Introduction

Post-stroke cognitive impairment (PSCI) is frequently found following stroke. More than 70% of stroke survivors suffered from PSCI of various severities, ranging from mild to severe impairment, within the first year [1,2]. This impairment disturbs daily life activities, cognition, and executive functions, which in turn worsens quality of life [2]. It is also recognized as an important burden which retards rehabilitation for performing activities of daily living [3,4]. Currently, the coexistence of stroke and stress is increasing due to the rising trends of exposure to both stroke [5] and stress [6]. Owing to the rising trends of stroke and stress exposure mentioned earlier, the prevalence of cognitive impairment following exposure to stroke under stress conditions is expected to continually rise and produces a significant negative impact in terms of socioeconomic burdens worldwide. Therefore, this problem should be considered, and prevention is also required. However, no current strategy directly targets this condition.

It has been found that one of the most common problems after stroke attack is dysphagia or difficulty in swallowing. The prevalence of this condition in stroke survivors is approximately 50% [7]. Most current drugs targeting PSCI that are available are in solid oral dosage form (SODF), which is not convenient for this condition because the crushing of medicine is required [8]. Thus, the key formulation factor in drug design for this group is to overcome this burden [9]. Therefore, a dosage form that does not need to be chewed or swallowed in order to ingest the drug is needed. An orodispersion film is an oral soluble thin film sheet that contains the drugs or supplement substance. It has a large surface area that permits a faster wetting, disintegration, and dissolution. Owing to its rapidly dissolvability, it can deliver an accurate amount of the active ingredient in an oral cavity, ease of use, and increased acceptability due to its ability to mask the undesired flavor of the active substance, it has gained much attention [9,10].

Currently, no drugs have been approved as the gold standard drug for treatment of PSCI. The most commonly used drugs are drugs used for treating other types of cognitive impairment, such as piracetam [11], and cholinesterase inhibitors, such as donepezil [12]. Most of them are synthetic drugs which can produce both advantages and side effects, whereas most herbal medicines produce synergistic actions on physiological systems, are less toxic, and are less expensive than synthetic drugs [13]. Ginger, or *Zingiber officinale*, and holy basil, or *Ocinum tenuiflorum*, are known for their anti-paralytic action, while the silkworm pupae protein, an insect protein commonly consumed in the North-Eastern region of Thailand since the ancient period, is rich in branched chain amino acids, which are essential for the restorative process following stroke [14,15]. In traditional folklore, most applications preferred polyherbal therapy over monoherbal therapy based on the concept of synergistic drug interaction [16]. Based on this concept, a combined extract of ginger and holy basil is developed, and our in vitro data also show that it has a combination index less than 1, which confirms the synergistic drug interaction, as shown in Supplementary Table S1. For the silkworm protein, which is rich in branched chain amino acids, the hydrolyfate form was selected because this form shows a better absorption than the concentrated protein [17]. Since the orodispersion film provides numerous advantages, we used it as a delivery tool. Starch derived from black rice, or *Oryza sativa* (black rice), which is safe and biodegradable [18] and rich in anthocyanin, a substance reputed for cognitive function enhancement [19], is selected and prepared as orodispersion film. Owing to the aforementioned factors, we developed an orodispersible film from black rice polymer loaded with silkworm pupae hydrolysate and the combined extract of holy basil and ginger, or “JP1”. It mitigates stroke severity under stress conditions, which produces more severe brain damage and dysfunction than stroke alone. It can also improve brain damage and brain dysfunction in ischemic stroke in combination with stress by decreasing oxidative stress, apoptosis, and inflammation [20]. Although the available drugs on the market such as piracetam can also improve the mentioned condition, they can also produce side effects such as anxiety, depression, insomnia, and irritability. In addition, the daily dose is high, around 2400 to 4800 mg, which increases the risk of renal toxicity [21]. Therefore, a novel strategy for preventing and attenuating PSCI is still required. Based on the fact that the inhibition of neuroinflammation and oxidative stress are responsible for the major underlying mechanisms of PSCI [22], the protective effect of JP1 against PSCI was examined. The combined extract of ginger and holy basil in JP1 has ginger extract as its main ingredient, so it should contain a high concentration of gingerol, the main ingredient in ginger extract [23]. A recent study demonstrated that the consumption of gingerol-enriched ginger rhizome can exert a positive modulation effect on gastrointestinal bacteria composition, giving rise to an improvement in indigestion symptoms [24]. Based on the beneficial effect of gingerol-enriched rhizome on the gut microbiota, the modulation effect of JP1 on gut microbiota had gained much attention. To date, no scientific evidence regarding the protective effect of JP1 against PSCI and the modulation effect of JP1 on gut microbiota is available. Therefore, we aimed to determine the mitigation effect and mechanism of action of JP1 on cognitive impairment following stroke under stress conditions. The possible underlying mechanisms via its direct effect on the brain area involving memory and cognitive function, such as the hippocampus and prefrontal cortex [25], and via an indirect effect through modulating the gut–brain axis function, such as increasing the density of probiotic bacteria such as *Lactobacillus* and *Bifidobacterium* spp., a bacterium group which can decrease oxidative stress, were explored.

## 2. Materials and Methods

### 2.1. Preparation of the Rice Polymer Loaded with Silkworm Pupae Hydrolysate and the Combined Extract of Ginger and Holy Basil (JP1)

JP1 was prepared as previously mentioned elsewhere [7]. In brief, the preparation of silkworm pupae hydrolysate was performed by using the enzymatic hydrolysis technique induced by ALCALASE^®^ Enzyme (*Bacillus licheniformis*) (Merck, Darmstadt, Germany) whereas the combined extract of ginger (*Zingiber officinale* L.) and holy basil (*Ocinum sactum* L.) was prepared from the mixture of hydroalcoholic extracts of both plants at a ratio which can provide synergistic interaction, and the black glutinous rice (*Oryza sativa* L. indica) was prepared as a polymer film. Then, this polymer was loaded with the combined extract of ginger and holy basil. It contained the polyphenolic compounds and flavonoids of JP1 at the concentrations of 117.00 ± 0.51 µg GAE/mg sample and 38.33 ± 1.00 µg QE/mg sample, respectively. The fingerprint chromatogram was previously shown [7] (Supplementary Figure S1). The concentrations of essential and non-essential amino acids were 9.53 and 52.53 mg/g sample, respectively. The three main essential amino acids are lysine, leucine, and phenylalanine, while glycine, proline, and glutamic acid are the main non-essential amino acids in JP1, as shown in Table 1.

### 2.2. Experimental Animals and Protocol

The experimental animals used in this study were male Wistar rats, weighing 200–250 g (n = 6/group), from the Northeast Laboratory Animal Center, Khon Kaen University, Thailand. All animals were housed in groups of 6 per cage under standard conditions (22 ± 2 °C, and 12:12 h light–dark cycle) and were allowed to access food and water ad libitum. All experimental protocols were approved by the Institutional Animal Care and Use Committee of Khon Kaen University, Thailand (IACUCKKU110/62). We randomly divided the animals into various groups as described in Table 2. In this study, we used 6 rats per group due to the sample size calculation (n = 5), and 20% of the total number was added to account for the loss induced by operation and suffocation, giving rise to 6 rats per group [13].

All oral treatments were performed 45 min prior to exposure to a 6 h immobilization stress. In this study, immobilization was induced by a restraint in a stainless-steel cage (5 cm diameter) with holes (0.5 cm diameter) around the cage for ventilation. Under this situation, rats could not move or turn around. The immobilization stress started at 9 A.M. and ran until 3 P.M. for 6 h a day [30,31] for 14 consecutive days. After 14 days of stress exposure, we induced the permanent occlusion of the right middle cerebral artery or induced the sham operation. Spatial and non-spatial memory assessments were performed by using the Morris water maze and object recognition tests, respectively, every 7 days throughout a 21-day study period after the operation. At the end of experiment, rats were sacrificed and acetylcholinesterase (AChE) activity, together with the neuron density in the prefrontal cortex and hippocampus, areas playing key roles in learning and memory [25], was determined. Owing to the pivotal role of the hippocampus in the interaction between the hippocampus and prefrontal cortex [32], inflammatory mediators such as interleukin-6 (IL-6), and tumor necrosis factor-alpha (TNF-α) [33], apoptosis mediators such as caspase-3 [34], and extracellular signal-regulated kinase (ERK), an important signal molecule involving learning and memory [35], in the hippocampus were explored. Furthermore, serum cortisol was also investigated at the end of the experiment.

### 2.3. Permanent Occlusion of the Right Middle Cerebral Artery (MCAO)

To induce anesthetization, pentobarbital sodium at a dose of 50 mg/kg BW was administered to each rat via an intraperitoneal route. Following this step, a longitudinal incision of a length of 1.5 cm was made at the midline of the ventral cervical skin. Then, the right common carotid artery (CCA), right external carotid artery (ECA), and right internal carotid artery (ICA) were carefully isolated from the vagus nerve and the adjacent tissues. A small incision was made to insert a silicone-coated monofilament nylon (number 4-0) from the lumen of CCA into the ICA, approximately 17–18 mm from bifurcation. Then, the cervical skin was sutured. After the operation, each rat was taken care of carefully until it recovered from anesthesia. Then, it was returned to the cage [36].

### 2.4. Behavioral Assessments

#### 2.4.1. Morris Water Maze Test

The Morris water maze test, a well-known and validated method, was used for assessing spatial memory. In brief, rats were trained to memorize the location of an immersed platform in 4 quadrants of a bathtub covered with non-toxic powder by using the external cues. The time each rat spent finding the location of the platform and climbing onto the platform was monitored and regarded as escape latency. The rat was re-exposed to the test again 24 h later, but the immersed platform was removed. The swimming time of the rat in the quadrant which previously contained an immersed platform was recorded as retention time [37].

#### 2.4.2. Novel Object Recognition Test

In this study, the novel object recognition test was used to assess non-spatial learning and memory in experimental rats. The experiments were conducted in a black open field apparatus (80 cm long × 50 cm high × 60 cm wide). The task procedure consisted of 3 sessions. During the T1 session, the rat was placed in the object recognition task containing two identical sample objects (A + A) for 3 min. Then, the rat was removed from the task and administered the assigned substances. During the T2 session, which was performed 30 min after administering the substances, the rat was exposed to an object recognition task containing two objects, but one sample was replaced by a novel or unfamiliar object (different size, shape, and color) (A + B). During the T3 session rat was re-exposed to the same task (one familiar, or A, and one novel object, or C), but the assessment was performed at 6 h after the substance administration. Each session was performed for 3 min, and the exploration time of the objects were recorded. Results was calculated and expressed as a novel object ratio (NOR) using the following equation:

NOR = (T_novel_ − T_familiar_)/(T_novel_ + T_familiar_)

where T_novel_ = time spent exploring the novel object and T_familiar_ = time spent exploring the familiar object [38,39].

#### 2.4.3. Locomotor Activity

The assessment of locomotor activity was performed by using an open field apparatus (90 × 70 cm). Each rat was placed at the center of the equipment. The number of times that each rat entered the center square of the open field chamber or crossed within a 5 min exploration time was recorded. Moreover, the number of exploratory activities, including licking, rearing, and grooming, were also observed [40].

### 2.5. Assessment of Oxidative Stress Status

The oxidative stress status of the brain tissues was determined by using malondialdehyde (MDA) level, with the activities of scavenging enzymes including superoxide dismutase (SOD), catalase (CAT), and glutathione peroxidase (GSH-Px) as indices. Firstly, hippocampi were isolated and homogenized with 0.1 M phosphate-buffered saline (1:50 W/V). Then, they were centrifuged at 3000× *g* at 4 °C for 15 min. The supernatant of tissue homogenates was collected and the protein concentration was determined using a Thermo Scientific NanoDrop 2000c spectrophotometer (Thermo Fisher Scientific, Wilmington, DE, USA) at the wavelength of 280 nm.

The level of malondialdehyde (MDA) was determined by using the thiobarbituric acid reaction method. Briefly, 100 μL of tissue homogenate was mixed with a reaction mixture containing 100 μL of 8.1% sodium dodecyl sulfate (Sigma-Aldrich, St. Louis, MO, USA), 750 μL of 20% acetic acid (Sigma-Aldrich, USA), 750 μL of 0.8% thiobarbituric acid (Sigma-Aldrich, USA), and 300 μL of distilled water. Then, the mixture was heated at 95 °C for 60 min. After cooling, 2500 μL of a mixture of n-butanol (Merck, Darmstadt, Germany)–pyridine (Merck, Germany) (15:1) and 500 μL of distilled water were added. Following this process, a 4000 rpm centrifugation was performed for 10 min. The separated butanol layer was collected, and absorbance was measured at 532 nm via a spectrophotometer. 1,1,3,3-tetramethoxy propane (Sigma-Aldrich, USA) at concentrations ranging between 0 and 15 μM were used as a standard in this study. Results were expressed in terms of nmol/mg protein [41].

Superoxide dismutase (SOD) activity was measured based on the inhibition rate of cytochrome C by the superoxide radical. Briefly, a cocktail solution consisting of 57 mM potassium phosphate buffer pH 7.8 (Sigma-Aldrich, USA), 0.1 mM ethylenediaminetetraacetic acid (EDTA) (Sigma-Aldrich, USA), 10 mM cytochrome C, and 50 μM xanthine (Sigma-Aldrich, USA) was prepared. Then, 200 μL of the cocktail solution and 20 μL of xanthine oxidase solution (0.90 mU/mL, Sigma-Aldrich, USA) were mixed with 20 μL of superoxide dismutase standard (Sigma-Aldrich, USA) or 20 μL of sample. Absorbance at 415 nm was measured using microplate reader (iMark™ Microplate Absorbance Reader). SOD activities were expressed as units/mg protein [42].

The activity of the CAT enzyme was monitored based on the ability of an enzyme to break down H_2_O_2_. Briefly, 10 μL of samples or standard was mixed with 50 μL of 30 mM hydrogen peroxide in 50 mM phosphate buffer, pH 7.0) BDH Chemicals Ltd., London, UK) for one minute. Then, the reaction was stopped by adding 25 μL of 5N sulfuric acid solution (Sigma-Aldrich, USA). Following this step, 150 μL of 0.005N 5 mM potassium permanganate (Sigma-Aldrich, USA) was added to the mixture and allowed to react with the excess peroxide. Then, absorbance at 490 nm was measured. A standard calibration curve was prepared by using the CAT enzyme (Sigma-Aldrich, USA) at concentrations ranging from 0 to 100 units/mL. The standard curve was plotted against the catalase activity. Results were expressed in units/mg protein [43].

The glutathione peroxidase (GSH-Px) enzyme was indirectly measured by a coupled reaction with glutathione reductase. In brief, 20 μL of sample or standard was mixed with 10 μL of 1 mM dithiothreitol (DTT) (Sigma-Aldrich, USA (in 6.67 mM potassium phosphate buffer (pH 7), 10 μL of 50 mM glutathione solution (Sigma-Aldrich, USA), and 100 μL of 30% hydrogen peroxide (BDH Chemicals Ltd., UK). At the end of a 5 min incubation period, 10 μL of 10 mM 5,5-dithiobis-2-nitrobenzoic acid (DTNB ((Sigma-Aldrich, USA (was added, and absorbance was measured at 412 nm. The standard calibration curve was prepared by using the GSH-Px enzyme (Sigma-Aldrich, USA) at various concentrations ranging from 0 to 5 units/mL. Results were expressed as units/mg protein [42].

### 2.6. Western Blot Assessments

In this study, the Western blot analysis was conducted to determine the expressions of interleukin-6 (IL-6), tumor necrosis factor-α (TNF-α), nuclear factor kappa-B (NF-kB), caspase-3, and extracellular signal-regulated kinase 1/2 (ERK1/2) in the hippocampus. The hippocampi were isolated and homogenized with the reagent containing mammalian protein extraction solution (M-PER; Pierce Protein Biology Product, Rockford, IL, USA) and protease inhibitor cocktail (Sigma-Aldrich, USA) at a ratio of 1:10 W/V. Then, they were subjected to a 12,000× *g* centrifugation at 4 °C for 10 min, and the supernatant was collected to determine the protein concentration using a Thermo Scientific NanoDrop 2000c (Thermo Fisher Scientific, Wilmington, DE, USA). Samples were diluted in Tris-Glycine SDS-PAGE loading buffer (Bio-Rad, Hercules, CA, USA) and heated at 95 °C for 10 min. Then, 20 µL of the sample, and 3 µL of biotinylated broad-range molecular protein marker (Vivantis, Shah Alam, Malaysia) were loaded and separated using 10% sodium dodecyl sulfate-polyacrylamide gel electrophoresis (SDS-PAGE). Following this step, the gel was transferred to a nitrocellulose membrane by using an electroblotting process at 70 Volts for 90 min. At the end of the mentioned process, the membranes were removed and washed with 0.05% TBS-T 3 times for 5 min each. Then, the membranes were incubated in the blocking reagent containing 5% nonfat-milk in 0.1% TBS-T for 1 h at room temperature. At the end of the incubation, the membranes were washed with 0.05% TBS-T 3 times for 5 min each and incubated at 4 °C overnight with one of the following antibodies: anti-IL-6 antibody (Abcam, Cambridge, MA, USA; dilution 1:1000), anti-TNF alpha antibody (Abcam, USA; dilution 1:1000), anti-NF-kB p65 antibody (Abcam, USA; dilution 1:2000), anti-caspase-3 (Abcam, USA; dilution 1:1500), anti-phospho-Erk1/2 (Thr202/Tyr204) (Abcam, USA; dilution 1:2000), oranti-Erk1/2 (Abcam, USA; dilution 1:1000). After the incubation with the antibody, the membranes were washed and incubated with anti-rabbit IgG HRP-linked antibody (Cell Signaling Technology, Danvers, MA, USA; dilution 1:2000) at room temperature for 1 h. Protein band density was detected by using Immobilon ECL Ultra Western HRP Substrate (Merck, Germany). The analysis was performed by using ImageQuant TL v.7.0 image analysis software (GE Healthcare, Piscataway, NJ, USA). The results were presented as the mean of band density relative to control (beta actin; Abcam, USA; dilution 1: 2000) band density.

### 2.7. Assessment of AChE Activity

The determination of AChE was conducted by the colorimetric method. Briefly, a mixture containing 20 μL of sample solution, 200 μL of 0.1 mM phosphate buffer (pH 8.0) (Sigma-Aldrich, USA), and 10 μL of 0.2 M DTNB (5,5′-dithio-bis-(2-nitrobenzoic acid)) (Sigma-Aldrich, USA) was mixed and incubated at room temperature for 5 min. Then, 10 µL of 15 mM acetylcholine thiochloride (ACTI) (Sigma-Aldrich, USA) was added and incubated at room temperature for 3 min. Absorbance was measured at 412 nm using microplate reader (iMark™ Microplate Absorbance Reader). The activity of AChE was calculated according to the following equation:

AChE activity = (ΔA/1.36 × 10^4^) × 1/(20/230)C

where ΔA = the difference of absorbance/minute and C = protein concentration of brain homogenate. Results were expressed as nmol/min.mg protein [39,44].

### 2.8. Determination of Corticosterone Level

On day 21 after MCAO induction, blood was collected from each animal and kept on ice. It was centrifuged at 2000× *g* at 4 °C for 15 min. The obtained serum was used for the measurement of corticosterone level using the Corticosterone ELISA kit (Abcam, UK). The process was conducted according to the brochure guidance. The result was expressed as ng/mL.

### 2.9. Determination and Enumeration of Lactobacillus spp. and Bifidobacterium spp.

A fecal sample was collected from each rat on day 7, day 14, and day 21 after MCAO induction to determine the enumeration of *Lactobacillus* spp. and *bifidobacterium* spp. First, 1 g of fecal sample was diluted in 9 mL of phosphate buffer solution (pH 7.4). Then, tenfold serial dilutions (ranging 10^−2^ to 10^−8^) were prepared. An aliquot of samples from each concentration at a volume of 100 mL were dropped onto a specific agar (MRS agar, pH 5.4) plate containing 1% CaCO_3_. Then, they were spread on the spread plate using the sterile spreader and incubated at 37 °C for 48 h in anaerobic conditions (in anaerobic jars or W-Zip standing pouch). After incubation, plates were removed from the incubator and the number of colonies growing on the plates were counted [45].

### 2.10. Histological Study

The brains were exposed to a transcardial perfusion with fixative solution containing 4% paraformaldehyde (Sigma-Aldrich, USA) in 0.1 M phosphate buffer, pH 7.4, overnight at 4 °C. Then, they were infiltrated with 30% sucrose (Merck, Germany) solution for 72 h at 4 °C. Serial sections of tissues were cut frozen on a cryostat (Thermo Scientific™ HM 525 Cryostat) at 20 μm thick. All sections were placed on slides coated with 0.3% aqueous solution of gelatin containing 0.05% aluminium potassium sulfate (Sigma-Aldrich, USA). The brain section was immersed in 0.1% cresyl violet (Sigma-Aldrich, USA) for 2 min, rinsed with distilled water, and dehydrated through graded alcohols (70, 95, 100% 2×) (RCI LabScan, Thailand). The sections were cleared with xylene for 3 min (2 times) and mounted using DPX mountant (Merck, Germany) [42]. The evaluation of neuron density in the hippocampus (3.72 mm posterior to bregma, ±2.2 mm laterally, and 3.4 mm dorsal–ventrally) and prefrontal cortex (anteroposterior 2.5–4.5 mm, mediolateral 0.2–1.0 mm) from the rat brain atlas [42] was performed under an Olympus light microscope model BH-2 (Hachioji, Japan) at 40× magnification. Results were expressed as density of neurons per 255 µm^2^.

### 2.11. Statistical Analysis

Data are expressed as mean ± standard error of mean (SEM). Statistical significance was evaluated by using one-way analysis of variance (ANOVA) followed by Tukey’s HSD post hoc test. *p*-values < 0.05 were considered as statistical significance. All statistical data analyses were performed using SPSS version 28.0.1.0 (IBM Corp. Released 2021. IBM^®^ SPSS^®^ Statistics for macOS).

## 3. Results

### 3.1. Effect of JP1 on Memory

#### 3.1.1. Effect of JP1 on Spatial Memory

**Figure 1:**
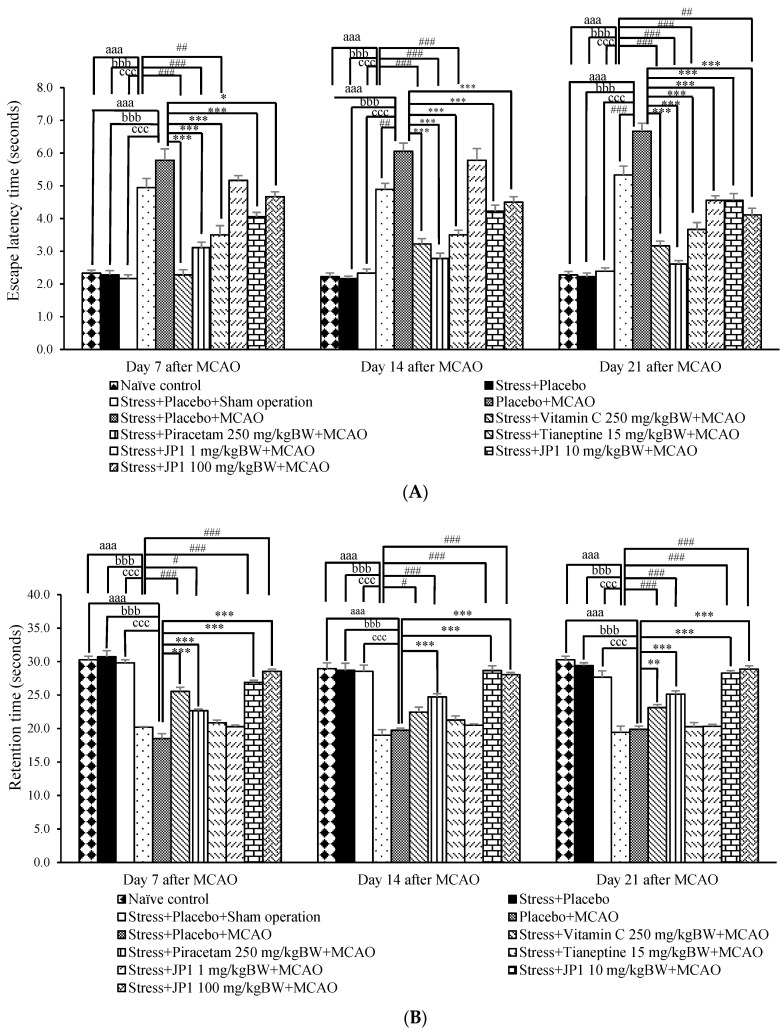
Escape latency and retention time of various groups throughout a 21-day study period after an operation: (**A**) escape latency time; (**B**) retention time. Data are presented as mean ± SEM (n = 6/group). ^aaa^ *p* value < 0.001 when compared to the naïve control group, ^bbb^ *p* value < 0.001 when compared to the stress + placebo-treated group, ^ccc^ *p* value < 0.001 when compared to the stress + placebo + sham operation-treated group, #, ##, ### *p* value < 0.05, 0.01 and 0.001, respectively, when compared to the placebo + MCAO-treated group, *, **, *** *p* value < 0.05, 0.01, and 0.001, respectively, when compared to the stress + placebo + MCAO-treated group.

Figure 1A demonstrates that when compared to the naïve control group, the stress + placebo-treated group failed to show significant changes in escape latency throughout the study period. This suggests that stress and placebo exerted no significant effect on escape latency. The stress + placebo + sham operation-treated group also showed no significant change in escape latency throughout the 21 days after the sham operation when compared to the naïve control group and the stress + placebo-treated group. Therefore, the sham operation did not produce any significant change in escape latency. The placebo + MCAO-treated group displayed a significant elevation in escape latency (*p*-value < 0.001 compared to the naïve control group, *p*-value < 0.001 compared to the stress + placebo-treated group, *p*-value < 0.001 compared to the stress + placebo + sham operation-treated group). These data suggest that the elevation in escape latency observed in this group was due to MCAO. The present data also showed that the stress + placebo + MCAO-treated group showed increased escape latency throughout the study period (*p*-value < 0.001 compared to the naïve control group, *p*-value < 0.001 compared to the stress + placebo-treated group, *p*-value < 0.001 compared to the stress + placebo + sham operation-treated group). When compared to the placebo + MCAO-treated group, it was found that the significant increase in escape latency was observed on day 14 and day 21 after MCAO (*p*-value < 0.01 and *p*-value < 0.001, respectively). These data suggest that stress exposure significantly exacerbated an increase in escape latency or exacerbated an impairment in spatial memory. All positive control-treated groups used in this study (vitamin-C, piracetam, and tianeptine) attenuated an elevation in escape latency in the stress exposure and MCAO-treated groups throughout the 21 days after MCAO (*p*-value < 0.001 all, compared to the stress + placebo + MCAO-treated group). When compared to placebo + MCAO, vitamin C, piracetam, and tianeptine also showed a reduction in escape latency on day 7 (*p*-value < 0.001, 0.001, and 0.01, respectively), day 14 (*p*-value < 0.001 all), and day 21 after MCAO (*p*-value < 0.001 all). These data suggest that an elevation in escape latency induced by MCAO and stress might involve many pathways, including alterations of oxidative stress status and cerebral blood flow and an alteration in neurotransmitters regulation. Interestingly, JP1 at the doses of 10 and 100 mg/kg BW significantly attenuated the elevation in escape latency when compared to the stress + placebo + MCAO-treated group on day 7 (*p*-value < 0.001 and 0.05, respectively), day 14 (*p*-value < 0.001 all), and day 21 after MCAO (*p*-value < 0.001 all), whereas a low dose of JP1 (1 mg/kg BW) produced a significant mitigation effect on the elevation in escape latency induced by stress and placebo only on day 21 after MCAO (*p*-value < 0.001 compared to the stress + placebo + MCAO-treated group, *p*-value < 0.01 compared to placebo + MCAO).

The effect of JP1 on retention time assessed by using the Morris water maze test is shown in Figure 1B. The stress + placebo-treated group showed no significant change in retention time throughout the study period. This lack of change suggests that the stress and placebo used in this study showed no effect on retention time. No significant change in retention time was observed in the stress + placebo + sham operation-treated group throughout the study period. Therefore, sham operation also failed to exert a significant influence on retention time. At 7 days after MCAO, the placebo + MCAO-treated group and the stress + placebo + MCAO-treated group revealed a significant reduction in retention time (*p*-value < 0.001 and 0.01, respectively, compared to the naïve control group, *p*-value < 0.001 all compared to the stress + placebo-treated group, *p*-value < 0.001 all compared to the stress + placebo + sham operation group). These changes were also observed on day 14 after MCAO (*p*-value < 0.001 all compared to the naïve control group, *p*-value < 0.001 all compared to the stress + placebo group, *p*-value < 0.001 all compared to the stress + placebo + sham operation-treated group) and day 21 (*p*-value < 0.001 all compared to the naïve control group, *p*-value < 0.001 all compared to the stress + placebo group, *p*-value < 0.001 all compared to the stress + placebo + sham operation-treated group). These data suggest that the reduction in retention time observed in this study involved MCAO operation, and stress failed to exacerbate the mentioned changes. When compared to the stress + placebo + MCAO-treated group, the significant increase in retention time of the stress + MCAO + vitamin C-treated group was observed on day 7 and day 21 (*p*-value < 0.001 all), whereas the mentioned change was observed in the stress + piracetam + MCAO-treated group throughout the 21 days after MCAO (*p*-value < 0.001 all). When compared to placebo + MCAO, vitamin C and piracetam also mitigated the reduction in retention time on day 7 (*p*-value < 0.001 and 0.05, respectively), day 14 (0.05 and 0.001, respectively), and day 21 after MCAO (*p*-value < 0.001, all). Unfortunately, no significant modification effect on the change in retention time in the stress + placebo + tianeptine-treated group was observed throughout the study period when compared either to the stress + placebo + MCAO-treated group or to the placebo + MCAO-treated group. Therefore, these data suggest that the modulation of oxidative stress status and cerebral blood flow may be associated with the reduction in retention time induced by stress and MCAO. The significant modulation effect on the reduction in retention time induced by stress and MCAO in the stress + MCAO + JP1-treated group at the doses of 10 and 100 mg/kg BW were observed throughout the study period (*p*-value < 0.001 all compared to the stress + placebo + MCAO-treated group). Therefore, JP1 improved spatial memory followed ischemic stroke and stress exposure. High and medium doses of JP1 produced better effects than a low dose of JP1.

#### 3.1.2. Effect of JP1 on Non-Spatial Memory

In this study, we assessed the novel object test via T1, T2, and T3 trials. In the T1 trial, animals were allowed to gain familiarity with objects. T2 was designed to allow the animal to differentiate between familiar and unfamiliar objects, and it was performed 30 min after the administration of the tested substance. T3 was also performed for the same purpose as T2 but it was performed 6 h after the administration of the tested substance. The reason for the measurement at different timepoints is that we do not know the absorption time and time to reach the maximum peak (Tmax) at which the concentration of the active ingredient can reach a therapeutic level, as well as the clearance time. Assessment at different time points can provide approximate time for the substance to exert its action and show the duration of action.

**Figure 2:**
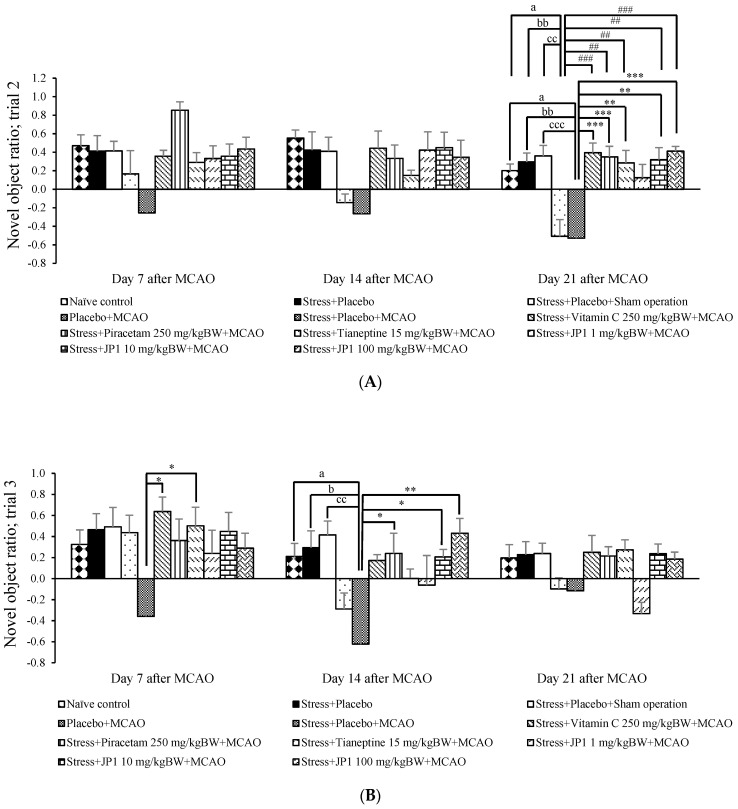
Novel object ratio (NOR) of various groups over a 21-day experimental period after an operation. (**A**) Novel object ratio (trial 2); (**B**) novel object ratio (trial 3). Data are presented as the mean ± SEM (n = 6/group). ^a^ *p* value < 0.05 when compared to the naïve control group, ^b, bb^ *p* value < 0.05 and 0.01, respectively, when compared to the stress + placebo-treated group, ^cc, ccc^ *p* value < 0.01 and 0.001, respectively, when compared to the stress + placebo + sham operation-treated group, ##, ### *p* value < 0.01 and 0.001, respectively, when compared to the placebo + MCAO-treated group, *, **, *** *p* value < 0.05, 0.01, and 0.001, respectively, when compared to the stress + placebo + MCAO-treated group.

Figure 2A reveals that no significant changes in NOR were observed in trial 2 or after 30 min of JP1 administration on day 7, day 14, or day 21 in the stress + placebo-treated group or the stress + placebo + sham operation-treated group. Therefore, stress, placebo, and sham operation did not produce significant change in NOR, which indicates non-spatial memory. The placebo + MCAO-treated group produced a significant reduction in NOR on day 21 (*p*-value < 0.05 compared to the naïve control group, *p*-value < 0.01 compared to the stress + placebo-treated group, *p*-value < 0.001 compared to the stress + placebo + sham operation-treated group). These data suggest that MCAO exerted an influence on NOR, which indicates non-spatial memory. The stress + placebo + MCAO-treated group also showed significantly decreased NOR on day 21 after MCAO (*p*-value < 0.05 compared to the naïve control group, *p*-value < 0.01 compared to the stress + placebo-treated group, *p*-value < 0.001 compared to the stress + placebo + sham operation-treated group). This change failed to show a significant difference when compared to the placebo + MCAO-treated group. This change suggests that stress did not exacerbate the impairment in NOR induced by MCAO. Vitamin C, piracetam, and tianeptine mitigated a reduction in NOR induced by MCAO (*p*-value < 0.001, 0.01, and 0.01, respectively, compared to the placebo + MCAO-treated group, and *p*-value < 0.001, 0.001, and 0.01, respectively, compared to the stress + placebo + MCAO-treated group) on day 21 after MCAO. The signification mitigation effect on NOR reduction induced by MCAO was also observed in the stress + MCAO + JP1-treated group at the doses of 10 and 100 mg/kg BW.

The effect of JP1 at the doses of 1, 10, and 100 mg/kg BW on NOR after 6 h of JP1 administration at 7, 14, and 21 days after the operation is shown in Figure 2B. The stress + placebo + MCAO-treated group showed significantly decreased NOR on day 14 after MCAO (*p*-value < 0.05 compared to naïve control, *p*-value < 0.05 compared to the stress + placebo-treated group, *p*-value < 0.01 compared to the stress + placebo + sham operation-treated group). On day 7 after MCAO, vitamin C showed a significant increase in NOR (*p*-value < 0.05 compared to the stress + placebo + MCAO-treated group), whereas piracetam produced a significant increase in NOR on day 7 and day 14 after MCAO (*p*-value < 0.05 all compared to the stress + placebo + MCAO-treated group). Both medium and high doses of JP1 increased NOR on day 14 after MCAO (*p*-value < 0.05, and 0.01, respectively, compared to the stress + placebo + MCAO-treated group). No significant change in NOR was shown in any groups on day 21 after MCAO. Therefore, our data revealed that JP1, an orodispersion film from rice polymer loaded with silkworm pupae hydrolysate and the combined extract of holy basil and ginger, could improve non-spatial memory as evaluated by object recognition test. The memory-enhancing effect on non-spatial memory was observed after the administration of medium and high doses of JP1, but no effect was observed after a low dose of treatment.

### 3.2. Changes in Neuron Density in the Prefrontal Cortex and Hippocampus

#### Changes in Neuron Density in the Hippocampus

**Figure 3:**
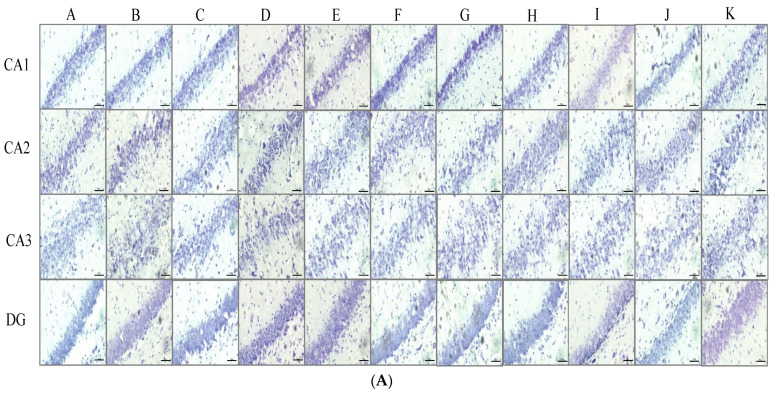

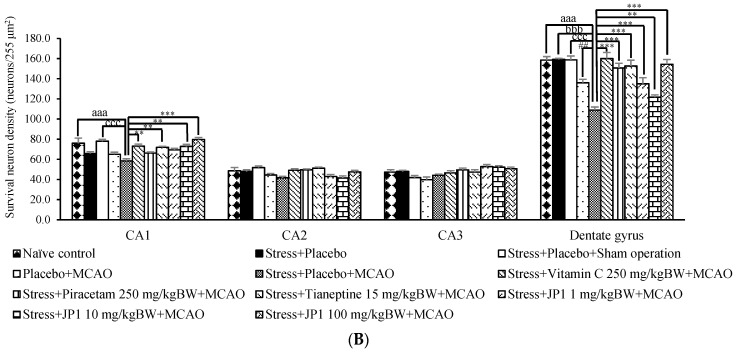
The density of survival neurons in various subregions of the hippocampus of different treatment groups. (**A**) Representative pictures of the survival neurons density in various subregions of the hippocampus. (**B**) Quantitative data on the survival neurons density in various subregions of the hippocampus. Data are presented as mean ± SEM (n = 6/group). ^aaa^ *p* value < 0.001 when compared to the naïve control group, ^bbb^ *p* value < 0.001 when compared to the stress + placebo-treated group, ^ccc^ *p* value < 0.001 when compared to the stress + placebo + sham operation-treated group, ## *p* value < 0.01 when compared to the placebo + MCAO-treated group, **, *** *p* value < 0.01 and 0.001, respectively, when compared to the stress + placebo + MCAO-treated group. Magnification 40× (scale bar = 50 μM). (A) The naïve control group, (B) the stress + placebo-treated group, (C) the stress + placebo + sham operation-treated group, (D) the placebo + MCAO-treated group, (E) the stress + placebo + MCAO-treated group, (F) the stress + vitamin C 250 mg/kg BW + MCAO-treated group, (G) the stress + piracetam 250 mg/kg BW + MCAO-treated group, (H) the stress + tianeptine 15 mg/kg BW + MCAO-treated group, (I) the stress + JP1 1 mg/kg BW + MCAO-treated group, (J) the stress + JP1 10 mg/kg BW + MCAO-treated group, (K) the stress + JP1 100 mg/kg BW + MCAO-treated group.

The current data show that no significant changes in neuron density in CA1, CA2, CA3, and DG of hippocampus were observed in the stress + placebo-treated group or the stress + placebo + sham operation-treated group. These data suggest that stress, placebo, and sham operation did not decrease neuron density in any areas of the hippocampus. Placebo + MCAO slightly decreased neuron density in CA1 and DG areas, but no significant change was observed. The decrease in neuron density in the placebo + MCAO-treated group suggested that MCAO may induce a slight neurodegeneration, and because of the small sample size and a variation, no significant change was observed. Stress + placebo + MCAO significantly decreased neuron density in CA1 (*p*-value < 0.001 compared to the naïve control group, *p*-value < 0.001 compared to the stress + placebo + sham operation-treated group) and DG (*p*-value < 0.001 compared to the naïve control group, *p*-value < 0.001 compared to the stress + placebo-treated group, *p*-value < 0.001 compared to the stress + placebo + sham operation-treated group). When compared to placebo + MCAO, it was found that stress + placebo + MCAO significantly decreased neuron density in DG (*p*-value < 0.01). Vitamin C and tianeptine produced a significant mitigation effect on the reduction in neuron density in CA1 and DG (*p*-value < 0.01 all, and *p*-value < 0.001 all compared to the stress + placebo + MCAO-treated group), whereas piracetam significantly mitigated the reduction in neuron density only in the DG of rats subjected to stress exposure and MCAO (*p*-value < 0.001 compared to the stress + placebo + MCAO-treated group). A low dose of JP1 mitigated the reduction in neuron density only in DG (*p*-value < 0.001 compared to the stress + placebo + MCAO-treated group), while the medium and high doses of JP1 significant increased neuron density in CA1 and DG (*p*-value < 0.01 all, and *p*-value < 0.001 all compared to the stress + placebo + MCAO-treated group), as shown in Figure 3A,B.

**Figure 4:**
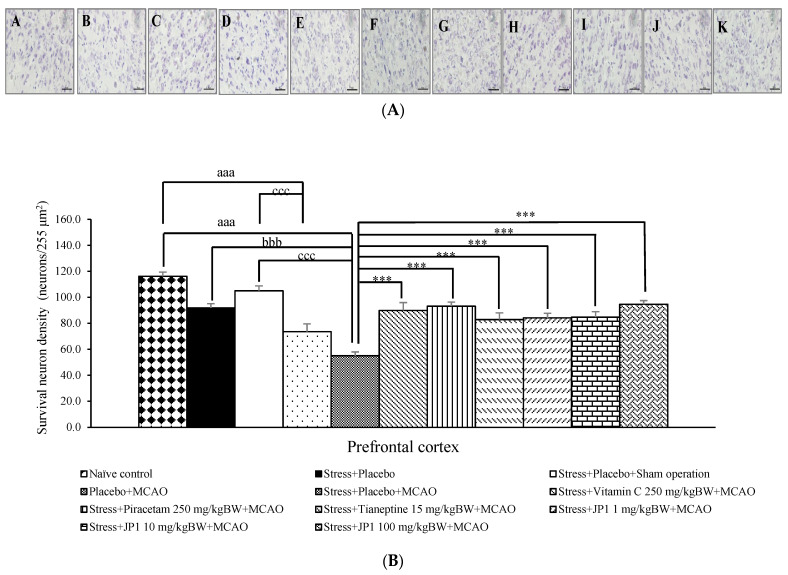
The density of survival neurons in the prefrontal cortex of various treatment groups. (**A**) Representative pictures of the survival neurons density in the prefrontal cortex. (**B**) Quantitative data on the survival neurons density in the prefrontal cortex. Data are presented as the mean ± SEM (n = 6/group). ^aaa^ *p* value < 0.001 when compared to the naïve control group, ^bbb^ *p* value < 0.001 when compared to the stress + placebo-treated group, ^ccc^ *p* value < 0.001 when compared to the stress + placebo + sham operation-treated group, *** *p* value < 0.001 when compared to the stress + placebo + MCAO-treated group-treated group. Magnification 40× (scale bar = 50 μM). (A) The naïve control group, (B) the stress + placebo-treated group, (C) the stress + placebo + sham operation-treated group, (D) the placebo + MCAO-treated group, (E) the stress + placebo + MCAO-treated group, (F) the stress + vitamin C 250 mg/kg BW + MCAO-treated group, (G) the stress + piracetam 250 mg/kg BW + MCAO-treated group, (H) the stress + tianeptine 15 mg/kg BW + MCAO-treated group, (I) the stress + JP1 1 mg/kg BW + MCAO-treated group, (J) the stress + JP1 10 mg/kg BW + MCAO-treated group, (K) the stress + JP1 100 mg/kg BW + MCAO-treated group.

The neuron density in the prefrontal cortex (PFC) of various treatment groups was also explored at the end of the study, and results are shown in Figure 4A,B. When compared to the naïve control group, there was no significant reduction in neuron density in the PFC of the stress + placebo-treated group and the stress + placebo + sham operation-treated group. Thus, stress, placebo, and sham operation failed to exert influence on the change in neuron density in the PFC. A significant decrease was found in neuron density in the PFC of the placebo + MCAO and the stress + placebo + MCAO-treated groups (*p*-value < 0.001 all compared to the naïve control group, *p*-value < 0.001 compared to the stress + placebo + sham operation-treated group). When compared to the stress + placebo-treated group, only the stress + placebo + MCAO-treated group showed a significant decrease in neuron density in the PFC (*p*-value < 0.001). The reduction in this parameter was mitigated by vitamin C, piracetam, tianeptine, and all doses of JP1 (*p*-value < 0.001 all compared to the stress + placebo + MCAO-treated group).

### 3.3. Change in Corticosterone Levels

**Figure 5:**
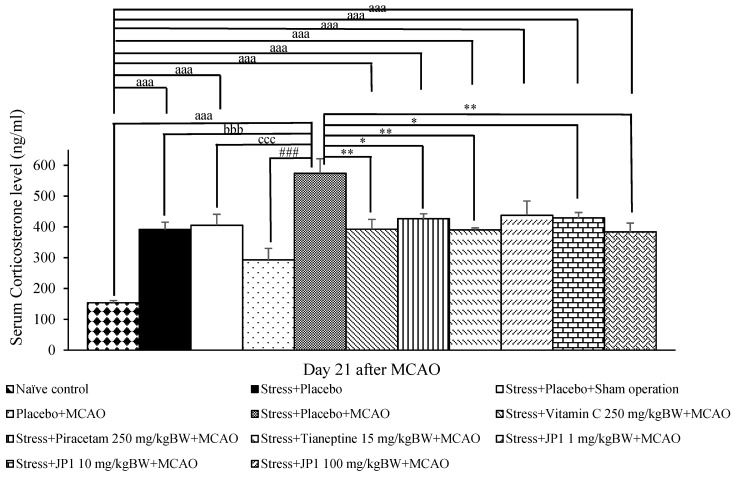
The effect of JP1 on serum corticosterone level after MCAO induction. Data are presented as mean ± SEM (n = 6/group). ^aaa^ *p* value < 0.001 when compared to the naïve control group, ^bbb^ *p* value < 0.001 when compared to the stress + placebo-treated group, ^ccc^ *p* value < 0.001 when compared to the stress + placebo + sham operation-treated group, ### *p* value < 0.001 when compared to the placebo + MCAO group-treated group, *, ** *p* value < 0.05 and 0.01, respectively, when compared to the stress + placebo + MCAO-treated group.

Figure 5 demonstrates that a significant elevation in corticosterone levels was observed in the stress + placebo-treated group and the stress + placebo + sham operation-treated group (*p*-value < 0.001 all compared to the naïve control group). These changes suggest that an elevation in corticosterone was associated with stress. When comparing between the stress + placebo-treated group and the stress + placebo + sham operation-treated group, no significant difference in corticosterone level was observed. This indicates that sham operation exerted no effect on this parameter. Placebo + MCAO slightly increased corticosterone level but no significant change was observed. Stress + placebo + MCAO produced a significant elevation in corticosterone level (*p*-value < 0.001 compared to the naïve control group, *p*-value < 0.001 compared to the stress + placebo-treated group, *p*-value < 0.001 compared to the stress + placebo + sham operation-treated group). This elevation in corticosterone in rats subjected to stress and MCAO was mitigated by vitamin C, piracetam, tianeptine, and JP1 at the doses of 10 and 100 mg/kg BW (*p*-value < 0.01, 0.05, 0.001, 0.05, and 0.01, respectively, compared to the stress + placebo + MCAO-treated group). When compared to the naïve control group, corticosterone levels in the mentioned groups were still higher than those in the naïve control group (*p*-value < 0.001 all). Therefore, the improvement in corticosterone levels mentioned earlier was still not fully repaired because the corticosterone levels were higher than those observed in the naïve control group. This change might occur because the rats were subjected to ischemic stroke induced by MCAO. Therefore, the suffocation induced by MCAO, such as neurological deficits including movement limitations like restraint and immobilization stress, still presented and could increase cortisol more than the baseline [46].

### 3.4. Acetylcholinesterase (AChE) Activity Changes in the Brain

**Figure 6:**
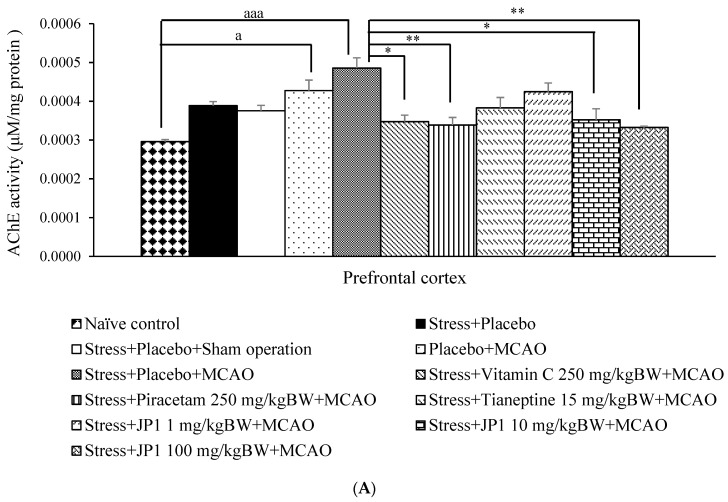

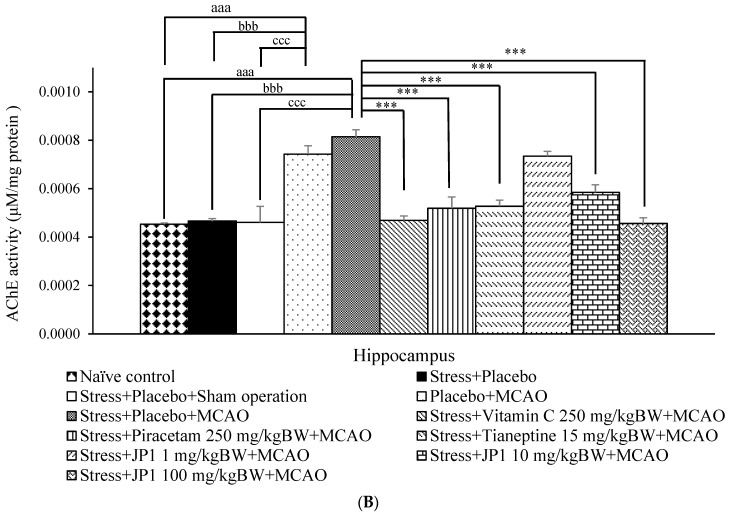
Acetylcholinesterase (AChE) activity of various treated rats at 21 days after MCAO. (**A**) AChE in the prefrontal cortex (**B**) AChE in the hippocampus. Data are presented as mean ± SEM (n = 6/group). ^a, aaa^ *p* value < 0.05 and 0.001, respectively, when compared to the control group, ^bbb^ *p* value < 0.001 when compared to stress + placebo, ^ccc^ *p* value < 0.001 when compared to stress + placebo + sham operation, *, **, *** *p* value < 0.05, 0.01, and 0.001 when compared to stress + placebo + MCAO.

Figure 6A reveals that both the stress + placebo and the stress + placebo + sham operation-treated groups failed to produce a significant change in AChE activity in the prefrontal cortex. These results suggest that stress, placebo, and sham operation did not exert a significant influence on AChE activity in prefrontal cortex area. The placebo + MCAO-treated group significantly increased AChE activity in the mentioned area (*p*-value < 0.05 compared to the naïve control group). Stress + placebo + MCAO produced a significant elevation in AChE activity (*p*-value < 0.001 compared to the naïve control group). Both vitamin C and piracetam significantly decreased AChE activity in stress-exposed rats subjected to MCAO (*p*-value < 0.05 and 0.01, respectively, compared to the stress + placebo + MCAO-treated group). Medium and high doses of JP1 also decreased AChE activity in the prefrontal cortex of stress-exposed rats subjected to MCAO (*p*-value < 0.05 and 0.01, respectively, compared to the stress + placebo + vehicle-treated group).

AChE activity in the hippocampus was also assessed, and results are shown in Figure 6B. When compared to the naïve control group, stress + vehicle, stress + vehicle + sham operation, and placebo + MCAO did not produce significant changes in AChE in the aforementioned area. The placebo + MCAO-treated group showed significantly increased AChE in the hippocampus (*p*-value < 0.001 compared to the naïve control group, *p*-value < 0.001 compared to the stress + placebo-treated group, *p*-value < 0.001 compared to the stress + placebo + sham operation-treated group). It was also found that stress-exposed rats that received placebo and were subjected to MCAO showed significantly increased AChE activity (*p*-value < 0.001 compared to the naïve control group, *p*-value < 0.001 compared to the stress + placebo-treated group, *p*-value < 0.001 compared to the stress + placebo + sham operation-treated group). An elevation in AChE activity in stress-exposed rats subjected to MCAO was mitigated by vitamin C, piracetam, tianeptine, and JP1 at the doses of 10 and 100 mg/kg BW (*p*-value < 0.001 all compared to the stress + vehicle + MCAO-treated group).

### 3.5. Alterations of Inflammatory and Apoptosis Mediators

**Figure 7:**
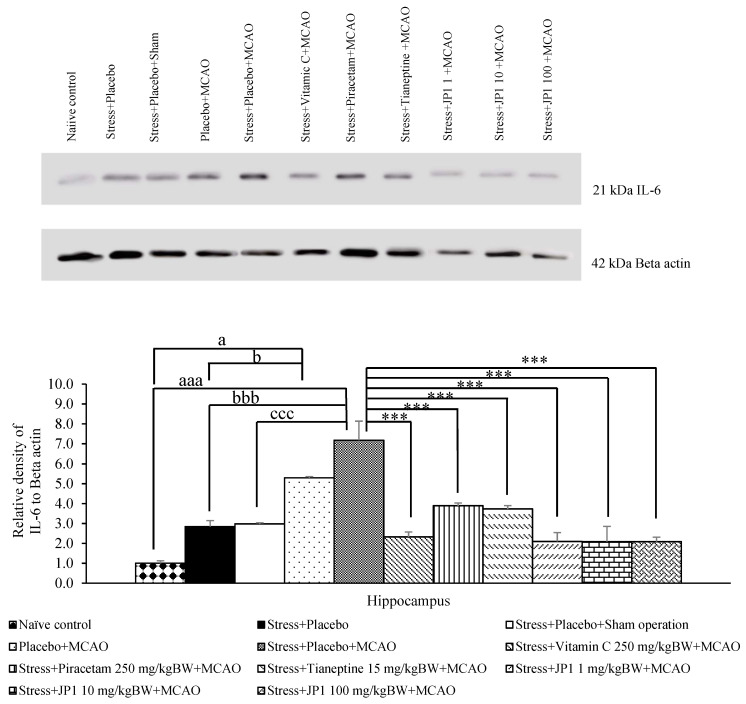
Interleukin-6 (IL-6) expression in the hippocampus of various treatment groups. Data are presented as mean ± SEM (n = 6/group). ^a, aaa^ *p* value < 0.05 and 0.001, respectively, when compared to the control group, ^b, bbb^ *p* value < 0.05 and 0.001, respectively, when compared to stress + placebo, ^ccc^ *p* value < 0.001 when compared to stress + placebo + sham operation, *** *p* value < 0.001 when compared to stress + placebo + MCAO.

Our results revealed marked changes in spatial memory, so we further explored the changes in inflammatory mediators such as IL-6, TNF-α, and caspase 3 in the hippocampus, an area with an important role in spatial memory. In addition, the mentioned markers were previously determined in our previous work. Figure 7 demonstrated that when compared to the naïve control group and stress + placebo, stress + placebo + sham operation produced no significant change in IL-6 in the hippocampus. Placebo + MCAO-treated rats also showed increased IL-6 (*p*-value < 0.05 compared to naïve control, *p*-value < 0.05 compared to the stress + placebo-treated group). The stress + placebo + MCAO-treated group produced a significant elevation in IL-6 in the mentioned area (*p*-value < 0.001 compared to the naïve control group, *p*-value < 0.001 compared to the stress + placebo-treated group, *p*-value < 0.001 compared to the stress + placebo + sham operation-treated group). These results suggest that stress, placebo, and sham operation produced no significant change in IL-6. MCAO produced a significant elevation in IL-6. This elevation was attenuated by vitamin C, piracetam, tianeptine, and all doses of JP1 in this study (*p*-value < 0.001 all compared to the stress + placebo + MCAO-treated group).

**Figure 8:**
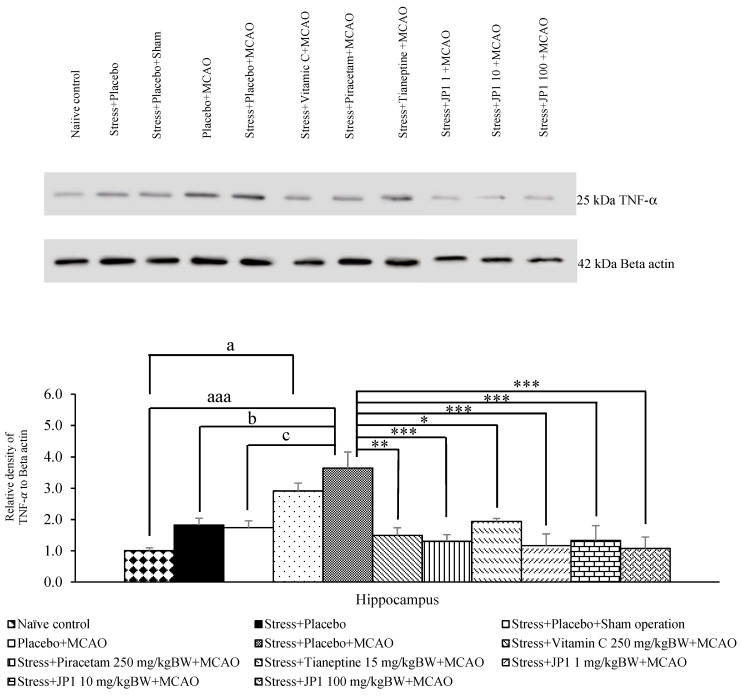
Tumor necrosis factor-alpha (TNF-α) expression in the hippocampus of various treated groups. Data are presented as mean ± SEM (n = 6/group). ^a, aaa^ *p* value < 0.05 and 0.001, respectively, when compared to the control group, ^b^ *p* value < 0.05 when compared to stress + placebo, ^c^ *p* value < 0.05 when compared to stress + placebo + sham operation, *, **, *** *p* value < 0.05, 0.01, and 0.001, respectively, when compared to stress + placebo + MCAO.

It was found that the stress + placebo and stress + placebo + sham operation produced no change in TNF-α in the hippocampus, as shown in Figure 8. Placebo + MCAO-treated rats showed significantly increased TNF-α in this area (*p*-value < 0.05 compared to the naïve control group). Stress-exposed rats that received placebo and were subjected to MCAO produced a significant elevation in TNF-α (*p*-value < 0.001 compared to the naïve control group, *p*-value < 0.05 compared to the stress + placebo-treated group, *p*-value < 0.05 compared to the stress + placebo + sham operation-treated group). However, this change was mitigated by vitamin C, piracetam, tianeptine, and JP1 at the doses of 1, 10, and 100 mg/kg BW (*p*-value < 0.001, 0.001, 0.05, 0.001, 0.001, and 0.001, respectively, compared to the stress + placebo + MCAO-treated group).

**Figure 9:**
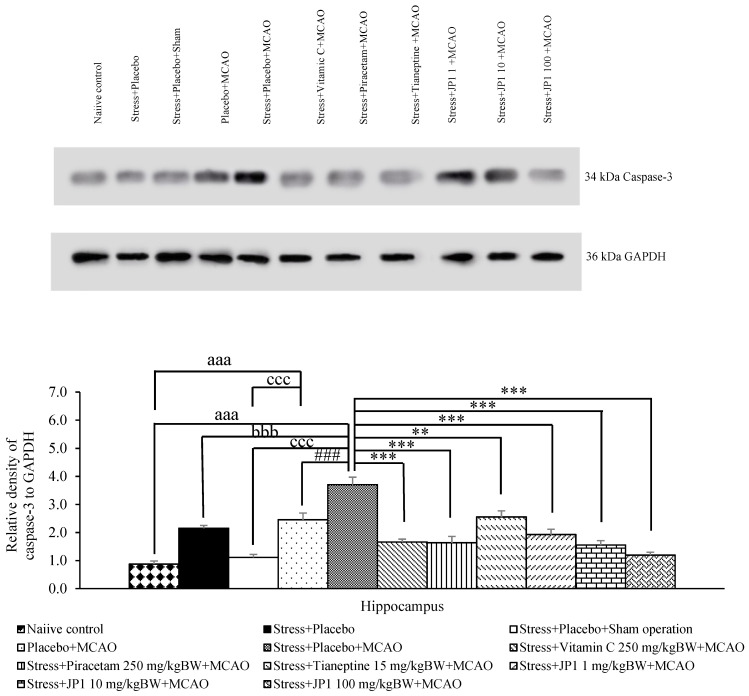
Effect of various treatments on the expression of caspase-3 in the hippocampus. Data are presented as mean ± SEM (n = 6/group). ^aaa^ *p* value < 0.001 when compared to the control group, ^bbb^ *p* value < 0.001 when compared to stress + placebo, ^ccc^ *p* value < 0.001 when compared to stress + placebo + sham operation, ^###^ *p* value < 0.001 when compared to placebo + MCAO, **, *** *p* value < 0.01 and 0.001, respectively, when compared to stress + placebo + MCAO.

Figure 9 reveals that when compared to the control group, stress + placebo and stress + placebo + sham operation failed to produce a significant change in caspase 3/GAPDH in the hippocampus. The placebo + MCAO-treated group showed a significant increase in the mentioned parameter (*p*-value < 0.001 compared to naïve control, *p*-value < 0.001 compared to the stress + placebo + sham operation-treated group). Stress-exposed rats that received placebo and were subjected to MCAO produced a significant increase in caspase3/GAPDH in the hippocampus (*p*-value < 0.001 compared to naïve control, *p*-value < 0.001 compared to the stress + placebo-treated group, *p*-value < 0.001 compared to the stress + placebo + sham operation-treated group). Vitamin C, piracetam, tianeptine, and JP1 at the doses of 1, 10, and 100 mg/kg BW significantly mitigated this elevation (*p*-value < 0.001, 0.001, 0.01, 0.001, 0.001, and 0.001, respectively, compared to the stress + placebo + MCAO-treated group).

### 3.6. Effect on Erk Signaling Pathway

**Figure 10:**
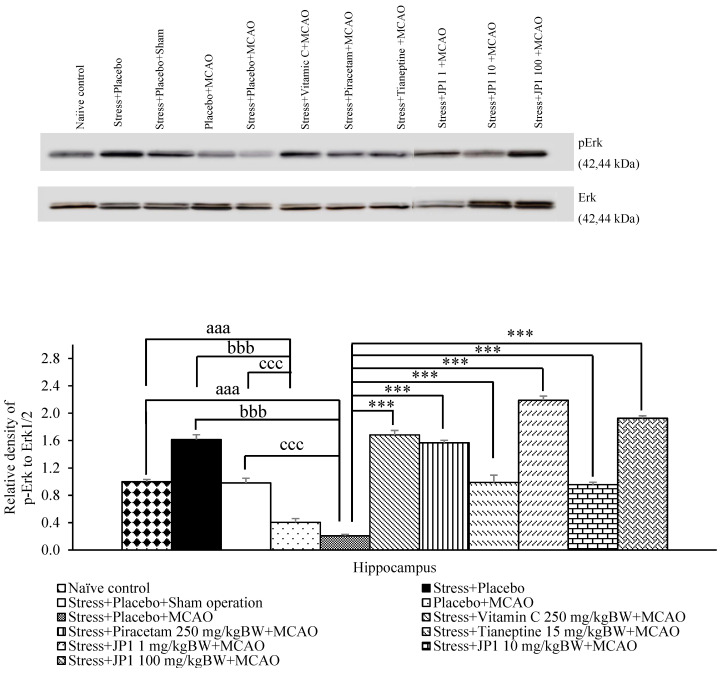
The effect of various treatments on the expression of pErk/Erk in the hippocampus. Data are presented as mean ± SEM (n = 6/group). ^aaa^ *p* value < 0.001 when compared to the control group, ^bbb^ *p* value < 0.001 when compared to stress + placebo, ^ccc^ *p* value < 0.001 when compared to stress + placebo + sham operation, *** *p* value < 0.001, respectively, when compared to stress + placebo + MCAO.

Owing to the role of Erk signaling pathway in learning and memory [35], the effect of JP1 on the mentioned pathway in the hippocampus was also explored. No significant change in this signal pathway was observed in the stress + placebo-treated group or the stress + placebo + sham operation-treated group. Placebo + MCAO-treated rats showed significantly reduced pErK1/Erk1/2 in the hippocampal region (*p*-value < 0.001 compared to the naïve control group, *p*-value < 0.001 compared to the stress + placebo-treated group, *p*-value < 0.001 compared to the stress + placebo + sham operation-treated group). Stress-exposed rats that received placebo and were subjected to MCAO produced a significant reduction in the mentioned parameter (*p*-value < 0.001 compared to the naïve control group, *p*-value < 0.001 compared to the stress + placebo-treated group, *p*-value < 0.001 compared to the stress + placebo + sham operation-treated group). However, this change was attenuated by vitamin C, piracetam, tianeptine, and all doses of JP1 used in this study (*p*-value < 0.001 all compared to the stress + placebo + MCAO-treated group), as shown in Figure 10.

### 3.7. Changes in Oxidative Stress Markers in the Hippocampus

Table 3 demonstrated that when compared to the naïve control group, no significant changes in MDA levels or the activities of SOD, CAT, and GSH-Px enzymes were observed in the hippocampus of the stress + placebo- and the stress + placebo + sham operation-treated groups. MCAO significantly increased MDA levels but decreased CAT activity in the mentioned area of rats that received placebo (*p*-value < 0.001 and 0.01, respectively, compared to the naïve control group). It was found that stress-exposed rats that received placebo and were subjected to MCAO also revealed an elevation in MDA levels together with the reduction in CAT activity (*p*-value < 0.001 and 0.01, respectively, compared to the naïve control group). Vitamin C, piracetam, tianeptine, and JP1 at the doses of 1, 10, and 100 mg/kg BW showed a mitigation effect on the elevation in MDA in stress-exposed rats that received placebo and were subjected to MCAO (*p*-value < 0.001 all compared to stress + placebo + MCAO). An elevation in CAT activity was observed in stress-exposed rats subjected to MCAO that received vitamin C, piracetam, and JP1 at the doses of 10 and 100 mg/kg BW (*p*-value < 0.01, 0.01, 0.05, and 0.05, respectively, compared to stress + placebo + MCAO). No significant changes in either SOD or GSH-Px were observed in this area of the stress-exposed rats subjected to MCAO.

### 3.8. Changes in the Amount of Lactobacillus spp. and Bifidobacterium spp. in the Feces of Various Treated Rats

**Figure 11:**
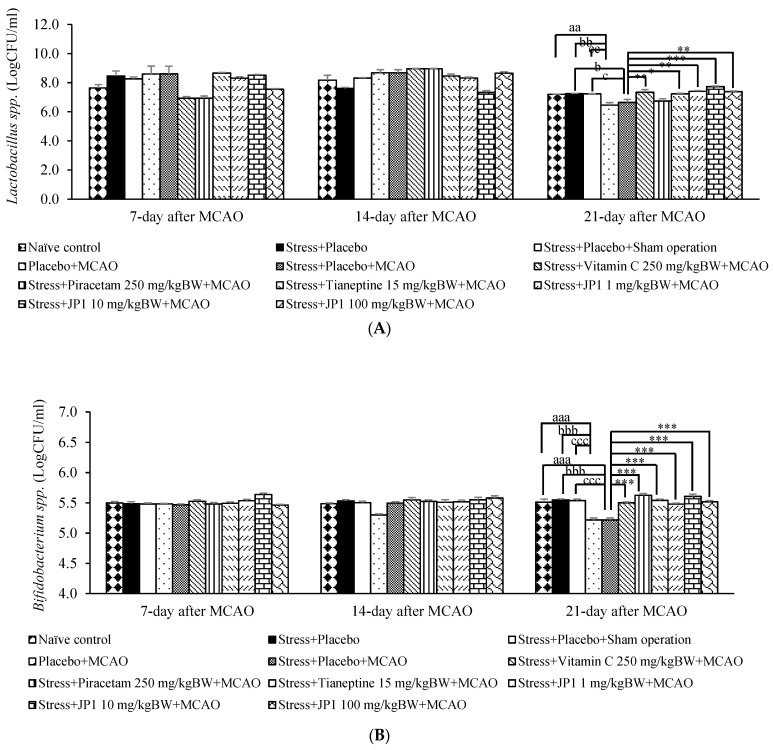
Effect of various treatments on amount of *Lactobacillus* spp. and *Bifidobacterium* spp. in the feces of various treated rats. (**A**) *Lactobacillus* spp. (**B**) *Bifidobacterium* spp. Data are presented as mean ± SEM (n = 6/group). ^aa, aaa^ *p* value < 0.01 and 0.001, respectively, when compared to the control group, ^b, bb, bbb^ *p* value < 0.05, 0.01, and 0.001, respectively, when compared to stress + placebo, ^c, cc, ccc^ *p* value < 0.05, 0.01, and 0.001, respectively, when compared to stress + placebo + sham operation, *, **, *** *p* value < 0.05, 0.01, and 0.001, respectively, when compared to stress + placebo + MCAO.

Figure 11A revealed that stress-exposed rats that received placebo and stress-exposed rats that received placebo and sham operation failed to show a significant change in the amount of *Lactobacillus* spp. in feces throughout the experimental period. On day 21 after MCAO, it was found that MCAO significantly decreased *Lactobacillus* spp. in feces of rats that received placebo (*p*-value < 0.01 compared to the naïve control group, *p*-value < 0.01 compared to the stress + placebo-treated group, and *p*-value < 0.01 compared to the stress + placebo + sham operation-treated group). Stress-exposed rats that received placebo and were subjected to MCAO also showed a reduction in this parameter (*p*-value < 0.05 compared to the stress + placebo-treated group, *p*-value < 0.05 compared to the stress + placebo + sham operation-treated group). This change was mitigated by vitamin C, tianeptine, and JP1 at the doses of 1, 10, and 100 mg/kg BW (*p*-value < 0.01, 0.05,0.01, 0.001, and 0.01, respectively, compared to the stress + placebo + MCAO-treated group).

A change in *Bifidobacterium* spp. density was also investigated, and the results are shown in Figure 11B. Stress-exposed rats that received placebo and stress-exposed rats that received placebo and sham operation did not produce a significant change in *Bifidobacterium* spp. density throughout the 21 days after MCAO. Placebo-treated rats subjected to MCAO showed a reduction in *Bifidobacterium* spp. density in feces at 21 days after MCAO (*p*-value < 0.001 compared to the naïve control group, *p*-value < 0.001 compared to the stress + placebo-treated group, and *p*-value < 0.001 compared to the stress + placebo + sham operation-treated group). Stress-exposed rats that received placebo and were subjected to MCAO demonstrated a significant reduction in *Bifidobacterium* spp. density at 21 days after MCAO (*p*-value < 0.001 compared to the naïve control group, *p*-value < 0.001 compared to the stress + placebo-treated group, and *p*-value < 0.001 compared to the stress + placebo + sham operation-treated group). Vitamin C, piracetam, tianeptine, and all doses of JP1 significantly attenuated the reduction in this parameter in stress-exposed rats subjected to MCAO (*p*-value < 0.001 all compared to the stress + placebo + MCAO-treated group).

## 4. Discussion

The present study clearly demonstrates that an orodispersion film derived from rice polymer loaded with silkworm pupae hydrolysate and the combined extract of ginger and holy basil improves both spatial and non-spatial memory in rats subjected to stress and cerebral occlusion in the right middle cerebral artery. It also decreases markers of oxidative stress, inflammation, and apoptosis but enhances pErk/Erk in the hippocampus. Furthermore, it also decreases serum corticosterone but increases the amount of *Lactobacillus* and *Bifidobacterium* spp. in the feces of experimental rats.

It is well established that both the hippocampus and prefrontal cortex play critical roles in the memory encoding and retrieval process [32]. It has been reported that memory deficit is associated with neuronal loss [47] and a reduction in cholinergic function [48,49] in the hippocampus and prefrontal cortex [50]. These changes correspond with the current results that demonstrate a neuronal loss in both the hippocampus and prefrontal cortex, a reduction in cholinergic function due to an elevation in AChE, and an impairment of both spatial and non-spatial memory types induced by MCAO. Numerous pathways such as oxidative stress, inflammation, and apoptosis [51,52] are associated with neurodegeneration. A large body of evidence has revealed that stroke injury increases excitotoxicity, leading to an elevation in intracellular calcium which in turn disturbs mitochondrial function. This change increases caspase-3, leading to apoptosis and memory impairment. In addition, the mitochondrial dysfunction also disturbs the function of the Erk signaling pathway, leading to a reduction in pErk/Erk1/2, giving rise to an elevation in caspase-3, and finally inducing apoptosis and memory impairment [53,54]. Furthermore, ischemic stroke also stimulate glial cells and macrophages, giving rise to the secretion of many inflammatory cytokines such as IL-6 and TNF-α, leading to inflammation and neuronal cell death [32]. Inflammation can increase oxidative stress, and oxidative stress can also increase inflammation [55], giving rise to neurodegeneration and memory impairment. Moreover, our results also reveal that ischemic stroke increases AChE activity, which corresponds with the results of a previous study. It has been proposed that this change also increases caspase 3 activity and apoptosis [56]. AChE is also associated with learning and memory. All changes, including oxidative stress, inflammation, apoptosis, Erk signaling, and AChE, play roles in neurodegeneration and memory impairment. These changes can be attenuated by JP1.

Beyond the mentioned mechanism, an increase in *Lactobacillus* and *Bifidobacterium* spp. densities also improves memory impairment [57]. Our results also demonstrate that MCAO decreases the amount of both *Lactobacillus* and *Bifidobacterium* spp. This change can be mitigated by JP1 at the dosage range used in this study. Gingerol, the main ingredient of the combined extract of ginger and holy basil [20], can increase the amounts of both of the mentioned species of bacteria [47]. It also exhibits antioxidant, anti-inflammation, and anti-apoptosis effects [48]. In addition, gingerol can suppress AChE [48,49] and improve the Erk signaling pathway [50], giving rise to memory improvement. Owing to these pieces of information, we suggest that the gingerol in JP1 may partly contribute to the memory-enhancing effect observed in this study. However, the effects of other ingredients and the interaction between the various ingredients present in JP1 still cannot be omitted.

The current study also demonstrates that stress increases serum corticosterone while MCAO alone failed to increase serum corticosterone. However, the coexistence of stress and MCAO can aggravate an elevation in serum corticosterone. In this study, the relationship between serum corticosterone and the density of neurons in the prefrontal cortex, and hippocampus and the closed relationship between serum corticosterone and memory failed to show a closed relationship. Therefore, the principal memory-enhancing effect of JP1 may not occur via the improvement of serum corticosterone. In addition, the reduction in serum corticosterone may also be attributed in part to gingerol [58]. The other ingredients and interactions among ingredients can also play roles.

No dose-dependent response of JP1 was observed in this study. The possible explanations for this may involve (1) the cognitive-enhancing mechanism of JP1 involving many pathways, so that no simple linear relationship between the concentration of JP1 and the observed parameters is present, and (2) the fact that JP1 contains numerous ingredients, and the increasing concentration of JP1 may also increase the possibility of producing a masking effect induced by other ingredients.

In this study, we used only an immobilization stress model, and this is a limitation of the study because different types of stress may involve different pathways and produce different results. Furthermore, we used only male rats as our experimental model, and the effect of stress in females may not be the same and so requires further investigation.

Although gingerol may contribute to the memory-enhancing effect, we still cannot ignore the effects of other substances, including some peptides and amino acids derived from the silkworm protein hydrolysate and some polyphenols such as anthocyanin derived from the rice polymer. In addition, the interaction among various ingredients still cannot be omitted. Therefore, exploration of the effect of each ingredient present in JP1 could also provide a better understanding regarding the possible active ingredient of JP1.

## 5. Conclusions

**Figure 12:**
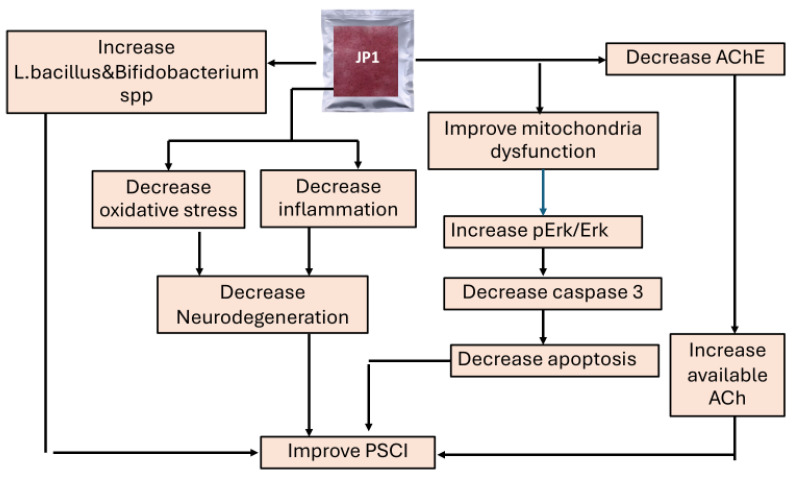
Schematic diagram illustrating the mechanisms of action of JP1.

This study demonstrates the cognitive-enhancing effect of JP1, a rice-derived orodispersion film loaded with silkworm hydrolysate and the combined extract of ginger and holy basil. It improves oxidative stress, inflammation, apoptosis, the Erk signaling pathway, and the cholinergic function in a combined stress and stroke condition as summarized in Figure 12. JP1 also decreases serum stress hormones such as corticosterone. Therefore, it may provide beneficial effects against stress-related disorders. However, the precise active ingredient and the mechanism of action require further investigation to provide better understanding.

## Figures and Tables

**Table 1:** The contents of essential and non-essential amino acids in JP1.

Contents of Amino Acids	Concentration (mg/g Sample)
**Essential amino acids (EAAs)**	
Threonine	0.78
Methionine	0.87
Phenylalanine	1.12
Histidine	0.51
Lysine	2.83
Valine	0.88
Isoleucine	0.55
Leucine	1.57
**Total EAAs**	9.11
**Non-essential amino acids (NEAAs)**	
Serine	0.94
Glycine	14.67
Glutamic acid	9.48
Proline	11.79
Cysteine	0.15
Alanine	5.75
Tyrosine	0.17
Arginine	5.93
Aspatic acid	3.53
Thyptophan	0.12
**Total NEAAs**	52.53

**Table 2:** Experimental animal groups, interventions, and descriptions.

Groups	Interventions	Descriptions
Group I	Naïve control group	Rats in this group were not subjected to any interventions.
Group II	Stress + placebo	All rats were subjected to placebo (the rice polymer film without silkworm pupae-derived protein hydrolysate and the herbal functional ingredients derived from ginger and holy basil) and induced immobilization stress by exposing them to a 6 h immobilization stress to disturb memory performance [26]. This group was used to determine the effect of stress and placebo treatment on memory.
Group III	Stress + placebo + sham operation	Rats were subjected to the same condition as mentioned in group II and received a sham operation by exposing the right middle cerebral artery without any occlusion. This group was designed to determine the effect of placebo and sham operation on memory in stress-exposed rats.
Group IV	Placebo + MCAO	Rats were subjected to a placebo treatment (without stress exposure) for 14 days before and 21 days after the operation to occlude the right middle cerebral artery (MCAO). This group was designed to determine the effect of placebo on memory in non-stress-exposed rats that were subjected to MCAO.
Group V	Stress + Placebo + MCAO	Rats were subjected to the conditions as mentioned for group III but they received MCAO instead of the sham operation. This group was designed to determine the effect of placebo on memory in stress-exposed rats that were subjected to MCAO.
Group VI	Stress + Vitamin C + MCAO	Rats in this group were subjected to the same conditions as mentioned for group V but vitamin C at a dose of 250 mg/kg BW was administered instead of placebo. This group was designed to determine the effect of vitamin C, a well-known antioxidant, on memory in stress-exposed rats that were subjected to MCAO.
Group VII	Stress + Piracetam + MCAO	Rats were subjected to the same conditions as mentioned for group VI but piracetam at a dose of 250 mg/kg BW was administered instead of vitamin C. This group was designed to determine the effect of piracetam, one of the standard neuroprotective drugs that can improve post-stroke cognitive impairment with vascular origin [27], on memory in stress-exposed rats subjected to MCAO.
Group VIII	Stress + Tianeptine +MCAO	The same conditions as mentioned for group VI were introduced, and tianeptine (15 mg/kg BW), a well-recognized antidepressant that blocks stress-induced alterations of neuronal morphology, synaptic plasticity, and memory loss induced by stress [28], was administered for 14 days before and 21 days after MCAO. This group was designed to determine the effect of tianeptine, a memory enhancer in stress-exposed rats via the glutamate pathway [29], in stress-exposed rats that received MCAO.
Group IX- Group XI	Stress + JP1 + MCAO	Rats in the mentioned groups were subjected to an intervention with JP1 at doses of 1, 10, and 100 mg/kg BW, respectively, for 14 days before and 21 days after Rt. MCAO. This group was designed to determine the effect of JP1 at the dosage range mentioned on memory in stress-exposed rats that received MCAO.

**Table 3:** Oxidative stress markers including MDA, SOD, CAT, and GSH-Px of various treatments in the hippocampus of various treated groups.

Groups	Hippocampus			
	MDA Level (ng/mg·Protein)	SOD Activity (units/mg·Protein)	CAT Activity (units/mg·Protein)	GSH-Px Activity (units/mg·Protein)
Naïve control	0.0112 ± 0.001 ^###,^***	50.59 ± 8.85	50.13 ± 5.87 ^##,^**	4.27 ± 0.38
Stress + Placebo	0.0198 ± 0.002	41.49 ± 1.12	24.75 ± 2.44	3.12 ± 0.42
Stress + Placebo + Sham operation	0.0186 ± 0.001	34.16 ± 3.49	25.88 ± 1.91	3.88 ± 0.48
Placebo + MCAO	0.0235 ± 0.001 ^aaa^	43.26 ± 3.16	26.3 ± 2.44 ^aa^	3.2 ± 0.38
Stress + Placebo + MCAO	0.0273 ± 0.001 ^aaa^	34.33 ± 1.22	25.98 ± 1.58 ^aa^	3.10 ± 0.37
Stress + Vitamin C 250 mg/kg BW + MCAO	0.0117 ± 0.001 ^##,^***	48.13 ± 6.32	50.01 ± 3.60 **	4.06 ± 0.40
Stress + Piracetam 250 mg/kg BW + MCAO	0.0136 ± 0.002 ^#,^***	44.97 ± 4.47	49.09 ± 7.25 **	3.65 ± 0.40
Stress + Tianeptine 15 mg/kg BW + MCAO	0.0144 ± 0.003 ^#,^***	42.24 ± 7.75	28.22 ± 2.44	3.54 ± 0.08
Stress + JP1 1 mg/kg BW + MCAO	0.0151 ± 0.001 ***	37.98 ± 3.19	40.29 ± 4.64	3.27 ± 0.26
Stress + JP1 10 mg/kg BW + MCAO	0.0141 ± 0.002 ^#,^***	39.78 ± 4.43	48.02 ± 6.24 *	3.27 ± 0.29
Stress + JP1 100 mg/kg BW + MCAO	0.0128 ± 0.002 ^##,^ ***	39.23 ± 1.22	48.34 ± 5.18 *	3.75 ± 0.26

Data are presented as mean ± SEM (n = 6/group). ^aa, aaa^ *p* value < 0.01 and 0.001, respectively, when compared to the control group, ^#^, ^##^, ^###^ *p* value < 0.05, 0.01, and 0.001, respectively, when compared to the placebo + MCAO group, *, **, *** *p* value < 0.05, 0.01, and 0.001, respectively, when compared to the stress + placebo + MCAO group.

## Data Availability

The data presented in this study are available on request from the corresponding author. The data are not publicly available due to privacy.

## Supplementary Materials

The following supporting information can be downloaded at: https://www.mdpi.com/article/10.3390/nu16234144/s1, Table S1: EC50 of antioxidant (DPPH, and FRAP assay), anti-inflammation (COX-2 inhibition), and the suppression effect of monoamine oxidase (MAO) of the ginger extract, holy basil extract, and the combined extract of ginger and holy basil extract; Figure S1: The fingerprint chromatogram of JP1 (100 mg/mL) derived by using HPLC analysis.
