# Supplementary material for: A Novel Supplement Consisting of Rice, Silkworm Pupae and a Mixture of Ginger and Holy Basil Improves Post-Stroke Cognitive Impairment
Source: Nutrients. 2024 Nov 29;16(23):4144. doi: 10.3390/nu16234144 (PMC11644478; doi:10.3390/nu16234144)

Table S1. EC50 of antioxidant (DPPH, and FRAP assay), anti-inflammation (COX-2 inhibition), and the suppression effect of monoamine oxidase (MAO) of the ginger extract, holy basil extract, and the combined extract of ginger and holy basil extract.

EC_{50} (μg/ml)	Ginger extract	Holy basil extract	Combined extract	Combination index (Type of interaction)
DPPH	221.16 ± 3.20	292.94 ± 0.80	63.12 ± 2.98	0.49 ± 0.01 (Synergism)
FRAP	457.09 ± 49.58	397.87 ± 7.09	212.51 ± 16.24	0.99 ± 0.01 (Synergism)
COX-II inhibition activity	152.88 ± 3.62	155.25 ± 2.78	34.52 ± 3.07	0.44 ± 0.02 (Synergism)
MAO inhibition activity	481.66 ± 13.98	537.71 ± 3.88	257.79 ± 37.02	0.95 ± 0.06 (Synergism)
α-amylase inhibition activity	2886.67 ± 23.33	1023.08 ± 13.92	673.02 ± 26.51	0.89 ± 0.01 (Synergism)

Figure S1. The fingerprint chromatogram of JP1 (100 mg/mL) derived by using HPLC analysis.

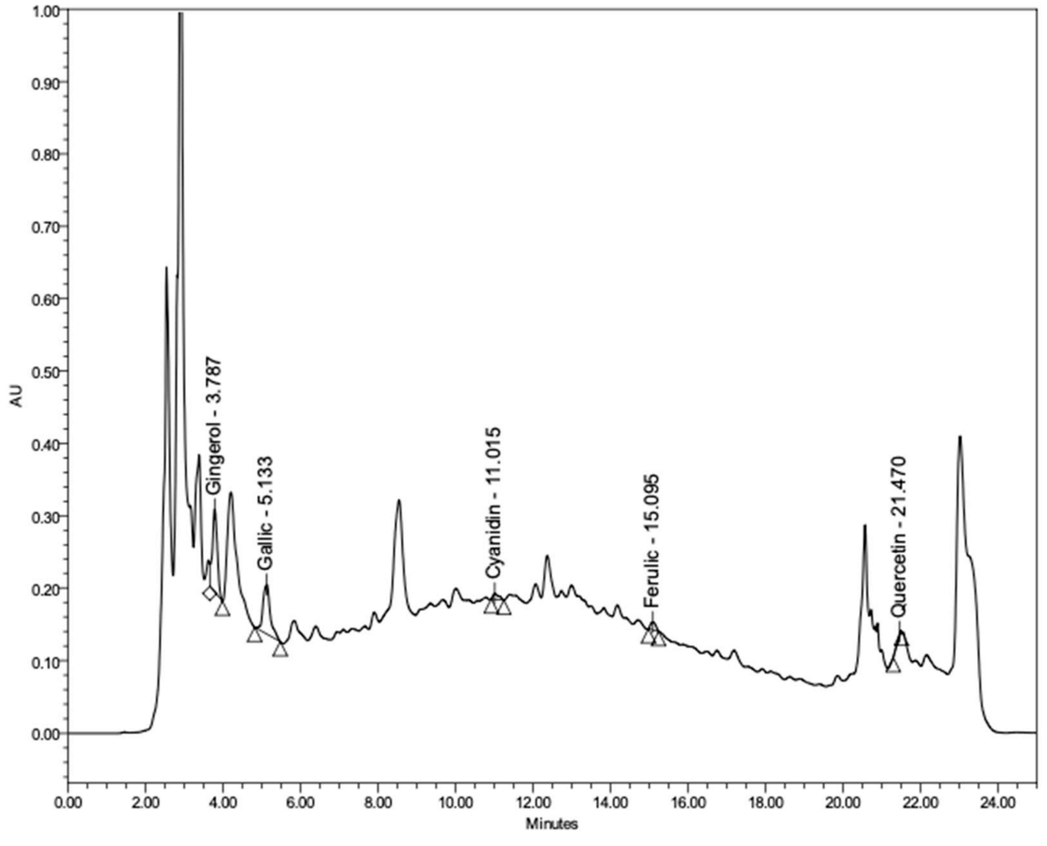

Supplement: Supplementary file 1 [file nutrients-16-04144-s001.zip › nutrients-3320605-supplementary.pdf]
